# Nitrogen-Doped Graphene via *In-situ* Alternating Voltage Electrochemical Exfoliation for Supercapacitor Application

**DOI:** 10.3389/fchem.2020.00428

**Published:** 2020-06-04

**Authors:** Mingjun Jing, Tianjing Wu, Yazheng Zhou, Xilong Li, Yong Liu

**Affiliations:** ^1^State Key Laboratory of Powder Metallurgy, Central South University, Changsha, China; ^2^Department of Chemistry, Xiangtan University, Xiangtan, China

**Keywords:** N-doped graphene, alternating voltage, electrochemical exfoliation, electrochemical performances, supercapacitors

## Abstract

Doping heteroatom, an effective way to enhance the electrochemical performances of graphene, has received wide attention, especially related to nitrogen. Alternating voltage electrochemical exfoliation, as a low cost and green electrochemical approach, has been developed to construct *in-situ* N-doped graphene (N-Gh) material. The N-Gh presents a much higher capacity than that of pure graphene prepared via the same method, which might be attributed to the introduction of nitrogen, which has much more effects and a disordered structure. As-prepared N-Gh exhibits a low O/C ratio that is helpful in maintaining high electrical conductivity. And the effects and disorder structure are also conductive to reduce the overlaps of graphene layers. A symmetric supercapacitor assembled with N-Gh electrodes displays a satisfactory rate behavior and long cycling stability (92.3% retention after 5,000 cycles).

## Introduction

Graphene exhibits exceptional electronic conductive ability and carrier mobility due to its unique quantum Hall effect on a honeycomb sp^2^ carbon lattice. Because of this, it became one of the most significant candidate materials for next-generation electronic and energy storage devices (Novoselov et al., 2004; Low et al., 2013; Gong et al., 2016). It is important to note that heteroatom-doped graphene might be better applied to supercapacitors through creating defects or embedding impurities. Among the various kinds of heteroatom-doped graphene materials, N atom is a general nominee because of its atomic size similarity to the carbon atom and unique valence electrons that generate a stable covalent bonds structure with adjacent C atoms (Low et al., 2013; Chaban and Prezhdo, 2015; Xu et al., 2018). Meanwhile, nitrogen atoms in N-doped graphene materials could become a redox active center, which might induce pseudocapacitance to increase the specific capacitance of materials (Luo et al., 2013; Yang et al., 2016). Hence, N-doped graphene or N-doped graphene-based composite materials are getting more and more attention.

*In situ* doping can be favorable for the formation of homogeneous doping (Qu et al., 2010; Yang et al., 2016). Some approaches have been developed to construct N-doped graphene. For example, N-graphene has been obtained via the chemical vapor deposition (CVD) method using a nitrogen-containing mixed gas (Bulusheva et al., 2017; Bu et al., 2018). Additionally, N-graphene also can be formed through the segregation growth approach (Zhang et al., 2011). However, most of these methods usually require expensive devices, multistep transfer processes, or result in a low yield. At present, developing a green and low cost method to prepare mass production of N-doped graphene is still a major challenge.

There are several methods for graphene preparation, such as chemical vapor deposition (Suk et al., 2011), epitaxial growth (Yang W. et al., 2013), mechanical exfoliation (Yi and Shen, 2015), chemical exfoliation (Liu and Wang, 2011), electrochemical exfoliation (Yang et al., 2015; Bakunin et al., 2019), and so on. It is worth noting that electrochemical exfoliation has been deemed a useful technique in producing high-quality graphene on a large scale owing to it being environmentally friendly, low cost, and requiring only simple operations (Low et al., 2013; Ejigu et al., 2019). Two electrochemical types, cathodic and anodic methods, have been mainly performed in electrochemical exfoliation with graphite as a working electrode. On the one hand, cathodic exfoliation with the graphite material as a cathode usually takes place in organic solvents (Yang Y. et al., 2013; Taheri Najafabadi and Gyenge, 2015). This process typically needs some intercalates cations from the electrolyte, such as alkylammonium salts, ionic liquids, molten salts, and so on. On the other hand, anodic exfoliation is typically carried out in aqueous electrolytes with graphite as an anode (Parvez et al., 2014). The main issue of this method is the requirement of a high positive voltage (about a few tens of volts) in the electrochemical process, which might induce structural degradation and oxidation of the carbon lattice. Recently, a novel alternating voltage electrochemical exfoliation approach has been applied to prepare few-layer graphene flakes in aqueous electrolytes (Jing et al., 2015). Compared with the direct voltage exfoliation, the degree of oxidation of the carbon lattice can be reduced in the alternative redox process. And the two graphite electrodes are used as working electrodes during the alternating voltage process, which is conducive to improving the exfoliation efficiency.

Electrolyte solution is one of the key factors in all types of electrochemical exfoliation methods. Li salts as cathodic exfoliation electrolyte organic solution can release Li^+^ ions that are reversibly intercalated into the inner spacing of graphite (Low et al., 2013). Aqueous H_2_SO_4_ solution as anodic exfoliation electrolyte system can produce oxygen radicals (O·) and hydroxyl (OH·) to open boundaries, which is helpful in facilitating ${\text{SO}}_{4}^{2-}$ intercalation, and then releasing SO_2_ to expand the interlayer distance of graphite (Yang et al., 2015). Inorganic salts aqueous solutions (such as (NH_4_)_2_SO_4_) as anodic exfoliation electrolyte system shows a similar electrochemical mechanism in H_2_SO_4_ solution, except for the existence of OH^−^ ions at the edge sites and grain boundaries (Zabihi et al., 2019). Aqueous NaOH/H_2_O_2_ solution has also been utilized during anodic electrochemical exfoliation, which could generate OH^−^ and ${\text{O}}_{2}^{2-}$ intercalation ions and appears to result in NaOH-induced electrochemical reduction of the oxygen functional groups of graphene (Rao et al., 2014). In addition, a range of reductive agents [such as sodium borohydride, (2,2,6,6-tetramethylpiperidin-1-yl]oxyl, ascorbic acid, and so on) as additives in electrolyte solution can improve the atomic ratio of C/O and control the exfoliation process (Rao et al., 2014). All these previous studies further indicate that the composition of electrolyte solution could mainly influence the functional groups, defects, atomic ratio of C/O, and yield of graphene. Based on the above analysis, *in-situ* nitrogen doping approaches might be achieved via adding nitrogen compounds into electrolyte solution during the electrochemical exfoliation of graphite. At present, few nitrogen compounds as additives (protic ionic liquid ethylammonium nitrate, ammonia, and natural biocompatible glycine) have been discussed to produce N-doped graphene in an anodic electrochemical exfoliation process (Usachov et al., 2011; Wang et al., 2012). But the related research is still poor, especially utilizing an alternating voltage electrochemical technique.

In this study, the alternating voltage electrochemical technique has been successfully applied to *in-situ* construct N-doped graphene (N-Gh) on a large scale by adding ammonium chloride salt to NaOH aqueous solution. Compared with as-prepared pure graphene (Gh) utilizing the same process, the N-Gh sample presents a larger size and much more effects. And the electrochemical properties of N-Gh have been investigated in three-electrode and two-electrode systems. The N-Gh sample reveals a satisfactory rate behavior and long cycling stability.

## Experimental Section

### Synthesis of N-Doped Graphene Electrode Material

Alternating voltage electrochemical exfoliation was fabricated with a two-electrode system utilizing two graphite rods as working electrodes. N-doped graphene (N-Gh) was prepared in 3 M NaOH and 3 M NH_4_Cl mixed aqueous solution. Both graphite rods were exfoliated via 5.0 V alternating voltage (50 Hz, YK-BP81005 regulator transformer) for 5 h. Then the as-exfoliated substrate was separated, and further washed using distilled water until the pH value was close to 7. At last, the N-Gh sample was obtained via the freeze-dried method. Pure graphene (Gh) was also put in 3 M NaOH aqueous solution under the same preparation conditions.

### Materials Characterization

The phase character of materials was studied via the X-ray diffractometer (XRD, Rigaku D/max 2550 VB^+^) from 10 to 80^o^ at 5° min^−1^ with Cu Kα radiation. The raman spectra of the as-prepared products were collected using a Raman spectrometer (HORIBA Labram HR Evolution). The morphology of the as-obtained materials were explored through scanning electron microscopy (SEM, JSM-6510LV) and transmission electron microscopy (TEM, JEM-2100F). Furthermore, the atomic arrangement was studied utilizing high-resolution transmission electron microscopy (HRTEM, JEM-2100F). Then, FT–IR spectrophotometer (AVTATAR, 370) was applied to test the surface functional groups of materials using KBr as a reference. Thermogravimetric analysis (TGA, NETZSCH STA449F3) from 25 to 900°C was utilized to measure the thermostability of materials with a heating rate of 5°C min^−1^ in air. Moreover, X-ray photoelectron Spectroscopy (XPS, ESCALab250) was tested to analyze the surface chemical composition of the as-obtained samples with C1s photoelectron peak at 284.6 eV as the reference.

### Electrochemical Measurement

Active materials, super P, and polyvinylidene fluoride (PVDF) were mixed in N-methyl-2-pyrrolidone (NMP) with a mass ratio of 80:10:10 to form a coating slurry. Then, the as-obtained slurry was pressed onto a round nickel foam current collector. Finally, the electrodes with a loading mass of about 1.5 mg cm^−2^ were formed through drying at 50°C in a vacuum overnight and pressing under a 10 MPa pressure. A classic three-electrode electrochemical test system was utilized to investigate the electrochemical characteristics of the as-prepared materials. This test system was composed of a working electrode, platinum foil counter electrode, and Hg/HgO reference electrode. It is worth noting that the working electrodes should be soaked in 2 M KOH aqueous solution for 12 h before the electrochemical test. Moreover, the symmetric supercapacitor was equipped with two as-prepared working electrodes using 2 M KOH as an electrolyte solution and a glassy fibrous material as a separator. And the related calculation for the symmetric supercapacitor is based on the total mass of active material. Cyclic voltammetry (CV) curves were measured on MULTI AUTOLAB M204 (MAC90086) at various scanning rates. Electrochemical impedance measurements (EIS) were tested on a CHI 660B electrochemical working station with the frequency range between 100 and 0.01 Hz. Galvanostatic discharge/charge files were investigated at room temperature on Land CT2001A battery cycler.

## Results and Discussion

### The Electrochemical Exfoliation via Alternating Voltage

**Scheme 1:**
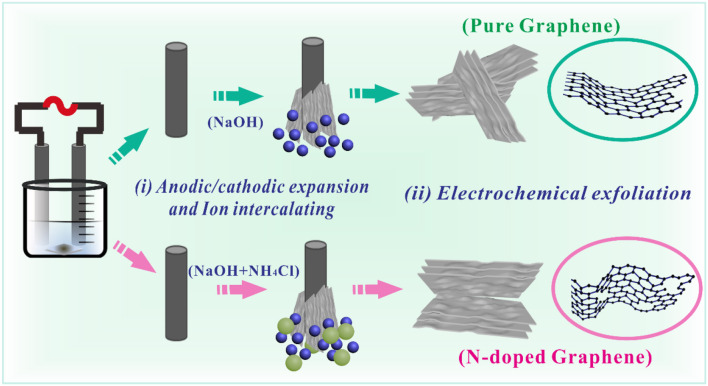
The electrochemical formation of pure Gh and N-Gh samples.

The electrochemical processes of the as-prepared Gh and N-Gh materials via alternating voltage electrochemical exfoliation have been displayed in Scheme 1. On the basis of previous reports (Wang et al., 2014; Jing et al., 2015), the surface of graphite electrodes were alternately oxidized and reduced during the electrochemical process of alternating voltage. Meanwhile, some cations and anions in the electrolyte solution can intercalate the layers of graphite to accelerate stripping speed. In detail, some defects and oxygen-containing functional groups on the surface of the graphite electrode have been induced during the anodic process. Then, some oxidized graphite was reduced via a cathodic reaction. In NaOH solution, the Na^+^ and OH^−^ can intercalate into the graphite layers. Certain amounts of hydrogen gas can be produced during the electrochemical process, which would promote the exfoliation rate of graphite. For the N-Gh sample, the addition of ${\text{NH}}_{4}^{+}$ and Cl^−^ ions might be conducive to exfoliate the graphite electrode through much more intercalation. Moreover, the Cl^−^ ions might be transformed into ClO^−^ or Cl_2_ during the electrochemical anodic process (Munuera et al., 2017). The related oxidation-reduction of ${\text{NH}}_{4}^{+}$ also took place during alternate anodic and cathodic reactions, which could generate C-NH_2_, -C-NH-C, and C-N-C_2_ functional groups. With the introduction of NH_4_Cl in the NaOH solution, the exfoliation rate can be effectively enhanced via alternating the voltage electrochemical process.

### The Microstructure, Morphology, and Composition of Samples

**Figure 1:**
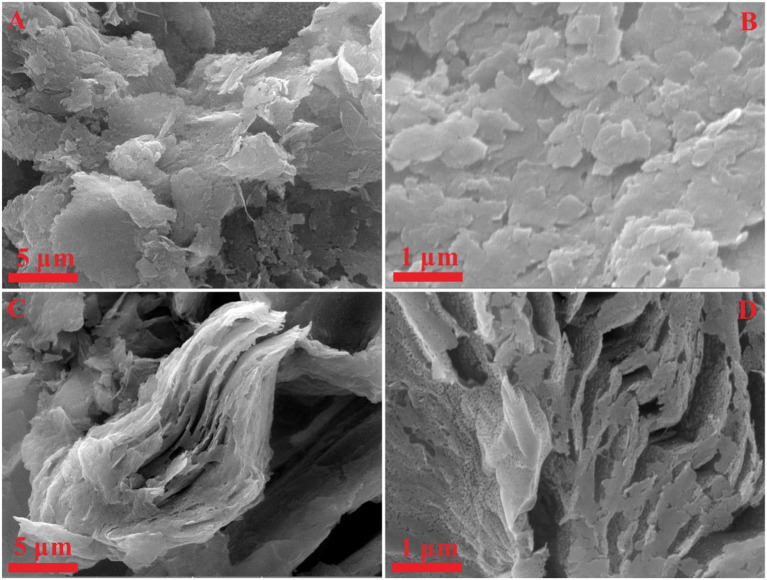
**(A,B)** SEM images of pure Gh. **(C,D)** SEM images of N-Gh sample.

**Figure 2:**
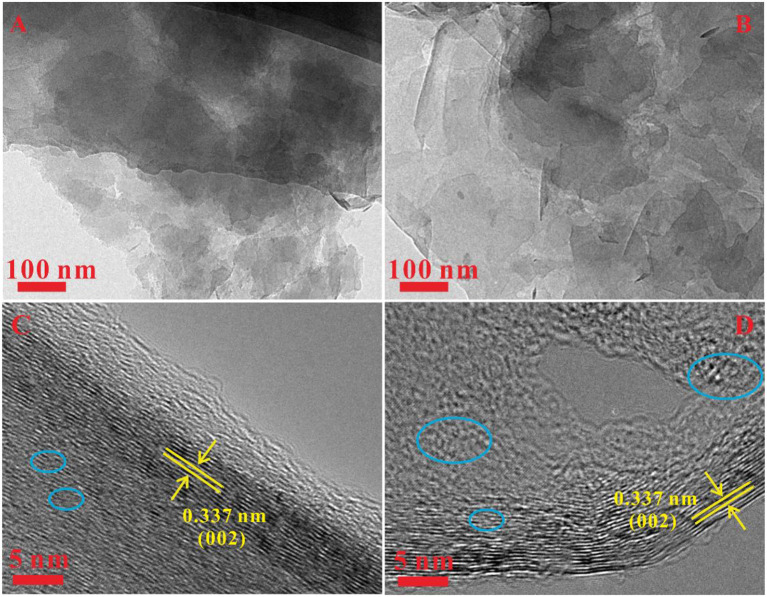
**(A,B)** TEM images of the pure Gh and N-Gh samples. **(C,D)** HRTEM images of pure Gh and N-Gh samples.

The SEM has been utilized to analyze the morphologies of the as-prepared Gh and N-Gh, which is shown in Figure 1. As displayed in Figures 1A,B, the pure Gh sample presents thin flakes with various sizes (0.5–5 μm^2^). In Figures 1C,D, the N-Gh displays porous thin sheets. It can also be seen that the size of N-Gh is much larger than that of pure Gh. Further, the characterization of the morphology has been measured using TEM, as shown in Figures 2A,C. These results reveal that the exfoliated Gh and N-Gh flakes typically feature some overlapping regions. Additionally, the HRTEM image of Gh (in Figure 2B) presents lattice spacing of 0.337 nm, corresponding to (002) plane of graphene (Yang et al., 2014). In Figure 2C, a few defect-free and disorder domains can also be found in the as-obtained pure Gh. While the N-Gh sample exhibits a much more disordered structure and obvious pore structure in Figure 2D. The N-Gh prepared by alternating voltage exfoliation presents with a larger size and more defects than those of pure graphene, which might be due to the fast stripping and N doping processes.

**Figure 3:**
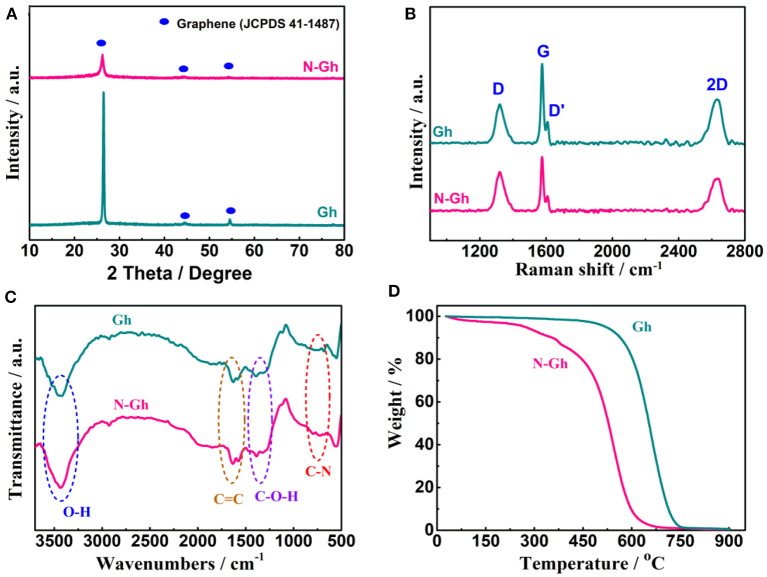
**(A)** XRD patterns of pure Gh and N-Gh samples. **(B)** Raman spectra of pure Gh and N-Gh samples. **(C)** FT-IR spectra of pure Gh and N-Gh samples. **(D)** TG curves of pure Gh and N-Gh samples.

Figure 3A presents the XRD powder pattern of pure Gh and N-Gh. The sharp peak of pure Gh at 26.4° is indexed as (002) crystal plane of graphene (JCPDS Card no.41-1487). This sharp peak illustrates that the Gh maintains a high degree of crystallization and electrical conductivity (Xu et al., 2015). According to the results of contrasting the curves of pure Gh and N-Gh, the peak intensity of the N-Gh sample is obviously weaker, which is consistent with the HRTEM conclusion. The order/disorder structures and defects characterization of the as-obtained materials have been further analyzed through Raman measurement, which is displayed in Figure 3B. The presence of G band at 1,577 cm^−1^ is related to E_2g_ symmetry phonon mode, corresponding to ordered in-plane sp^2^ carbon atoms (Wang et al., 2017). The D band and D' shoulder band are at about 1,321 and 1,621 cm^−1^, respectively, which belong to the disorder in the carbon hexagons and edge carbons (Deng et al., 2011). Moreover, the I_2D_/I_G_ and I_D_/I_G_ values of samples can typically reflect the number of layers and the degree of disorder structure, respectively (Soin et al., 2017). The I_2D_/I_G_ values of pure Gh and N-Gh are 61.9 and 62.7%, respectively, which could illustrate that the flakes of both samples have only a few layers. The I_D_/I_G_ value of N-Gh is 0.71, which is higher than that of pure Gh (0.57). This result again reveals that much more defects and disorder structure appear during the exfoliation process for N-Gh samples.

Furthermore, the surface functional groups of the as-obtained pure Gh and N-Gh have been detected via FT-IR spectrum analysis, as is displayed in Figure 3C. The large peaks at 3,432 cm^−1^ are related to O-H bending from H_2_O (Wang X. et al., 2018). The weak peaks from 1,639 to 1,579 cm^−1^ can be indexed to sp^2^-hybridized C=C stretching in plane vibrations, which reveals the presence of the π-conjugation structure (Wang X. et al., 2018; Lee et al., 2019). The weak peaks from 1,383 to 1,308 cm^−1^ correspond to oxygen-containing functional groups (C-O, C-OH, C-O-C) which illustrate the existence of a few hydroxyl/phenolic/alkoxy groups on the surface of the exfoliated Gh and N-Gh samples (Lee et al., 2019). A small peak at 728 cm^−1^ might correspond to C-N stretching (Islam et al., 2016), which indicates that N can be successfully doped *in-situ* during alternating voltage electrochemical exfoliation. In Figure 3D, the mass loss from 25 to 300°C of N-Gh might be mainly resulted from adsorbed water and coordinated water (Chen et al., 2020). Compared to the pure Gh, the TGA curve of N-Gh with the temperature from 300 to 700°C displays a quick downward trend, which illustrates the existence of much more defects and disorder structure in the as-obtained N-Gh sample (Xu et al., 2015).

**Figure 4:**
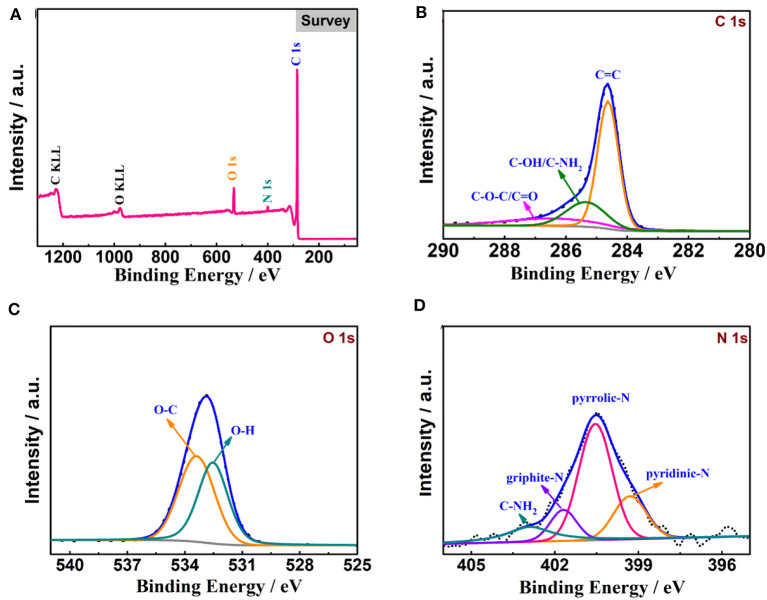
XPS analysis of N-Gh material: **(A)** Full spectrum, **(B)** C 1 s spectrum, **(C)** O 1 s spectrum, **(D)** N 1 s spectrum.

The XPS spectra of N-Gh are displayed in Figure 4. As shown in Figure 4A, the C, O, and N elements all lie in the as-prepared N-Gh sample. The N atom content is 4.5% and the O/C atom ratio in this sample is 0.09. Further, a dominant peak at 284.6 eV is shown in the high-resolution XPS spectrum of C 1s (Figure 4B), which is assigned to graphitic C=C species, and the other two weak peaks at 285.5 eV and 286.4 eV correspond to sp^3^ carbons (C-OH/C-NH_2_) and oxygen carbons (C-O-C/C=O), respectively (Wang et al., 2014). The O 1s spectrum presents two peaks at 532.1 and 533.7 eV in Figure 4C, which are related to O=C and O-C, respectively (Hou et al., 2015; Bulusheva et al., 2017). A few oxygen-containing functional groups in the N-Gh sample could be helpful to relieve overlapping. The N 1s spectrum (in Figure 4D) has been fitted into four peaks at 399.3 eV, 400.5 eV, 401.7 eV, and 402.9 eV, which are assigned to pyridinic nitrogen (19.8%), pyrrolic nitrogen (54.2%), graphite nitrogen (11.9%), and C-NH_2_ (14.1%), respectively (Lee et al., 2014; Hong et al., 2019). These results again confirm that N has been successfully doped in N-Gh, which might contribute to the improvement of electrochemical performances.

### The Electrochemical Properties of Samples Based on a Three-Electrode System

**Figure 5:**
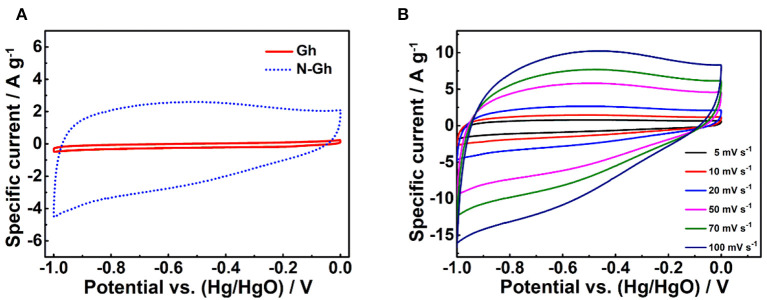
**(A)** The CV files of pure Gh and N-Gh samples at a 10 mV s^−1^ based on a three-electrode system. **(B)** The CV curves of the as-prepared N-Gh under various scan rates from 5 to 100 mV s^−1^ based on a three-electrode system.

The electrochemical performances of pure Gh and N-Gh samples have been firstly explored using CV tests in 2 M KOH electrolyte solution with the voltage range from −1.0 to 0 V based on a three-electrode system, which is shown in Figure 5A. It can be clearly seen that pure Gh and N-Gh samples display rectangular CV curves, suggesting obvious electric double-layer storage behaviors (Munuera et al., 2017). The curve area of N-Gh is much larger than that of the pure Gh sample, which indicates the doping of N can obviously improve the specific capacity. Furthermore, the CV measurements of N-Gh based on increased scan rates from 5 to 100 mV s^−1^ have been studied, as is revealed in Figure 5B. The intensities of CV files increased with the increased scan rates, yet the shapes of curves remained broadly stable (Zhu et al., 2018; Tang et al., 2019). This result reveals that N-Gh might present good electrochemical reversibility.

**Figure 6:**
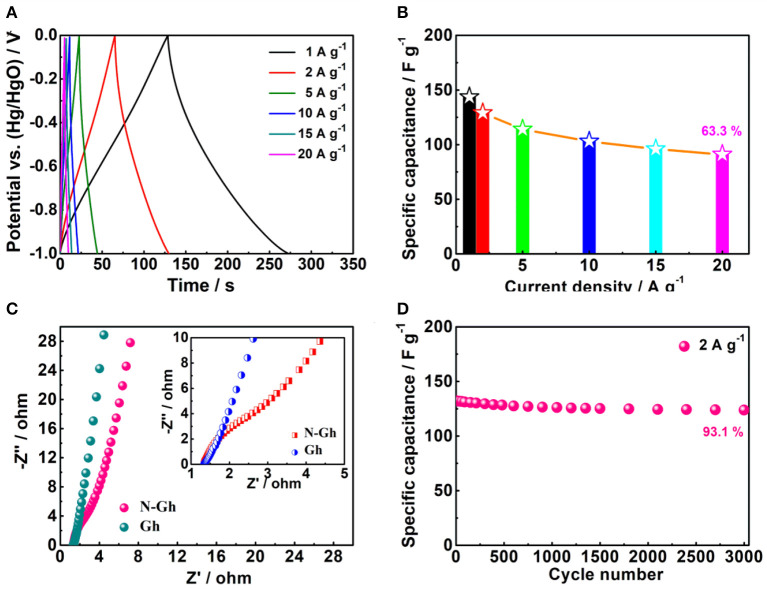
**(A)** Galvanostatic charge-discharge files and **(B)** Specific capacities of the as-prepared N-Gh at various current densities from 1 to 20 A g^−1^ based on a three-electrode system. **(C)** Impedance spectroscopy plots of Gh and N-Gh in a three-electrode system (The inset is a larger version of the impedance spectroscopy plots). **(D)** Cycling stability of N-Gh at 2 A g^−1^ based on a three-electrode system.

Then, the charge-discharge files of the as-prepared N-Gh electrode in 2 M KOH electrolyte solution at various current densities are displayed in Figure 6A. Based on the specific capacitance formula (C_s_ = It/mΔV, F g^−1^) (Wang Y. et al., 2018; Wei et al., 2019), the specific capacitances of N-Gh electrode at the current densities of 1, 2, 5, 10, 15, and 20 A g^−1^ are 143.6, 129.1, 114.2, 103.2, 96.5, and 91.5 F g^−1^, respectively, with high coulombic efficiency around 100%, which is displayed in Figure 6B. Compared with the specific capacitance at 1 A g^−1^, the capacity retention rate is up to 63.7% even at 20 A g^−1^. This high rate behavior might be related to the existence defects and doping N in the N-Gh sample. Moreover, the EIS curves of pure Gh and N-Gh electrodes in 2 M KOH aqueous solution at their open voltages are shown in Figure 6C. Both samples present very small semicircles in the high frequency and high slope in the low frequency region, suggesting the pure Gh and N-Gh electrodes display high electrical conductivity and ion diffusion. The cycling stability of N-Gh electrodes have been investigated, which is displayed in Figure 6D. After 3,000 charge/discharge cycles in KOH aqueous solution, the specific capacity retention of N-Gh is up to 93.1% at a current density of 2 A g^−1^. All these improved electrochemical performances could be derived from the structure of N-Gh sample with N doping, which has few effects and a disordered structure.

### The Electrochemical Performances of N-Gh Based on a Two-Electrode System

**Figure 7:**
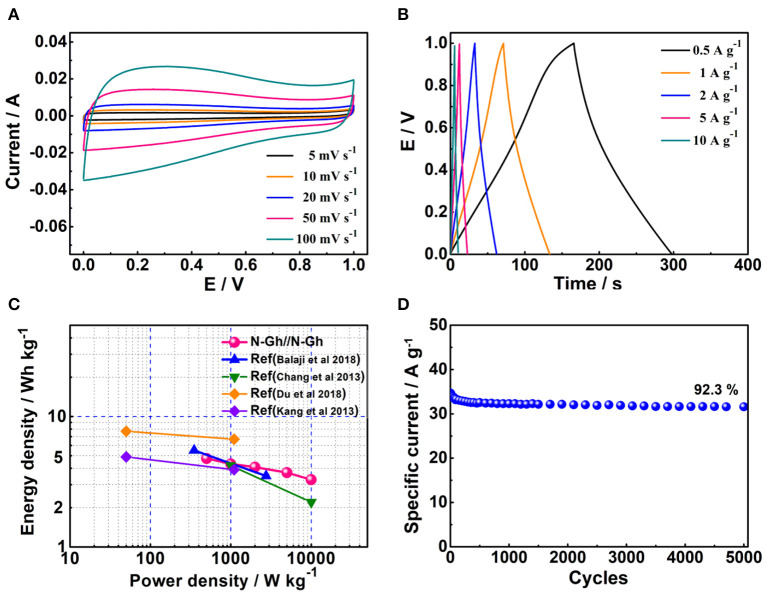
**(A)** The CV files of N-Gh at various scan rates from 5 to 100 mV s^−1^ based on a two-electrode system. **(B)** Galvanostatic charge-discharge files of N-Gh//N-Gh with the current densities range from 1 to 20 A g^−1^. **(C)** Ragone plots of N-Gh//N-Gh. **(D)** Cycling stability of N-Gh//N-Gh at 0.5 A g^−1^.

To better study the electrochemical capacity performances of the as-obtained N-Gh, the symmetric supercapacitor has been fabricated with two N-Gh electrodes. Figure 7A displays the CV curves of N-Gh//N-Gh symmetric supercapacitor under the potential voltage from 0 to 1 V at different scan rates. It can be clearly seen that the shape of files stays the same as the scan rate increases (from 5 to 100 mV s^−1^), indicating the N-Gh//N-Gh symmetric supercapacitor might display good electrochemical reversibility. The charge/discharge files of N-Gh//N-Gh symmetric supercapacitor at the current densities between 0.5 and 10 A g^−1^ are revealed in Figure 7B. The charge curves and discharge curves are almost symmetrical, revealing high columbic efficiency. Additionally, the specific capacities of the N-Gh//N-Gh symmetric supercapacitor have been calculated on the discharge files on the basis of the total mass of negative and positive electrode slices, as shown in Figure 7C. The specific capacities of the symmetric supercapacitor are 34.2, 31.2, 29.4, 26.7, and 24.2 F g^−1^ at current densities of 0.5, 1, 2, 5, and 10 A g^−1^, respectively. The energy density of this symmetric supercapacitor is 4.76 Wh kg^−1^ at a power density of 500 W kg^−1^, and the retention ratio is up to 68.7% at the power density of 10,000 W kg^−1^. Compared with some other N doped carbon-based materials (in Figure 7C) (Chang et al., 2013; Kang et al., 2013; Balaji et al., 2018; Du et al., 2018), the energy density and rate behavior of N-Gh//N-Gh is satisfactory. The cycling stability has been also investigated in Figure 7D, presenting with 92.3% retention of N-Gh//N-Gh initial specific capacity after 5000 cycles. The perfect rate behavior and cycling stability of the N-Gh//N-Gh symmetric supercapacitor further illustrates the as-prepared N-Gh might be a promising material for various kinds of composites in supercapacitors.

## Conclusion

In summary, the N-Gh sample has been prepared through an *in-situ* alternating voltage electrochemical exfoliation technique with the introduction of NH_4_Cl in to NaOH aqueous solution. The N chemical states in the N-Gh sample mainly present pyrrolic nitrogen. Compared with the as-obtained pure Gh sample, the N-Gh shows a larger size, much more effects, and a disordered structure. Additionally, the related electrochemical behaviors have been investigated in a three-electrode aqueous solution system, indicating that N-Gh displays a much higher specific capacity than that of pure Gh. Moreover, it also displays a good cycling stability and high rate behavior with 63.7% of the capacity retention rate even at a current density of 20 A g^−1^. All these good electrochemical characteristics of N-Gh could be ascribed to the doping N, the existence of effects, and disorder structure, which is conducive to producing faradaic pseudocapacitance and reducing overlapping layers of graphene. The results of the symmetric supercapacitor fabricated with two N-Gh electrodes further illustrate the satisfactory cycling stability with 92.3% retention of N-Gh//N-Gh initial specific capacity after 5,000 cycles. These insights illustrate that the N-Gh sample prepared via an *in-situ* alternating voltage approach could have promising applications to construct composites for enhanced supercapacitors.

## Data Availability Statement

All datasets generated for this study are included in the article/supplementary material.

## Author Contributions

MJ and TW designed and engineered the samples, performed the experiments, and wrote the paper. All authors contributed to performing the data analysis and general discussion.

## Conflict of Interest

The authors declare that the research was conducted in the absence of any commercial or financial relationships that could be construed as a potential conflict of interest.
